# Recent Advances in Responsive Microgels for Biomedical Application

**DOI:** 10.3390/bioengineering13060609

**Published:** 2026-05-23

**Authors:** Hongtao Zhang, Wenkai Zhang, Yongfeng Gao

**Affiliations:** 1 School of Chemistry and Chemical Engineering, Qinghai Normal University, Xining 810008, China; zhanght21@lzu.edu.cn; 2 Qinghai Women and Children’s Hospital, Xining 810001, China; 13897457701@163.com; 3 Department of Chemistry, University of Alberta, Edmonton, AB T6G 2G2, Canada

**Keywords:** stimuli-responsive microgels, controlled drug delivery, tissue engineering, imaging, diagnostic

## Abstract

Responsive microgels have emerged as a versatile class of soft materials for biomedical applications owing to their tunable physicochemical properties, high water content, and ability to respond dynamically to external and biological stimuli. This review summarizes recent advances in the design, synthesis, and biomedical utilization of responsive microgels, with a focus on their functional roles across key application domains. First, the fundamental principles governing microgel responsiveness and structure–property relationships are briefly introduced. The application of responsive microgels in controlled drug delivery is then discussed, highlighting stimulus-triggered release mechanisms, payload protection, and spatiotemporal control of therapeutic delivery. Advances in tissue engineering are reviewed with emphasis on microgel-based scaffolds, injectable constructs, and cell–matrix interactions that promote tissue regeneration. The use of microgels in biomedical imaging is examined, including their roles as contrast agents, signal amplifiers, and carriers for imaging probes. Finally, recent developments in microgel-enabled diagnostics are presented, showcasing their utility in biosensing, biomarker detection, and point-of-care platforms. The literature was selected based on the authors’ expertise, focusing on representative and recent studies, and identified through general academic databases and key references. Collectively, this review provides a comprehensive overview of the multifunctional capabilities of responsive microgels and discusses current challenges and future opportunities toward their clinical translation.

## 1. Introduction

Microgels are three-dimensional, crosslinked polymeric microparticles capable of absorbing water, deforming under mild stress, and encapsulating a wide range of therapeutic and diagnostic agents [1,2,3].Their highly tunable physicochemical properties—particle size, softness, porosity, degradability, and surface functionality—enable precise tailoring for biomedical use. In recent years, the convergence of polymer chemistry, microfabrication technologies, and bioengineering has accelerated the development of next-generation stimuli-responsive microgels, which undergo reversible and predictable physicochemical changes in response to environmental cues such as pH, temperature, ionic strength, enzymes, redox gradients, light, and magnetic fields [4,5,6]. These dynamic behaviors provide spatiotemporal control over microgel swelling, degradation, and cargo release, positioning responsive microgels at the forefront of emerging biomedical technologies.

The biomedical interest in responsive microgels stems from their ability to integrate both materials intelligence and biological relevance. Compared with bulk hydrogels, microgels offer faster response kinetics due to their high surface-area-to-volume ratio, improved injectability for minimally invasive administration, and the capacity to circulate or localize within tissues depending on design [7]. These features have driven rapid expansion of microgel platforms for targeted and controlled drug delivery, injectable scaffolds for regenerative medicine, immunomodulation, wound healing, biosensing, and theranostic systems. Moreover, advances in microfluidic synthesis, emulsion templating, and bioorthogonal crosslinking chemistries have enabled precise control over microgel architecture, allowing researchers to create multi-stimuli, anisotropic, and hybrid microgels that can mimic the complexity of biological microenvironments.

**Figure 1:**
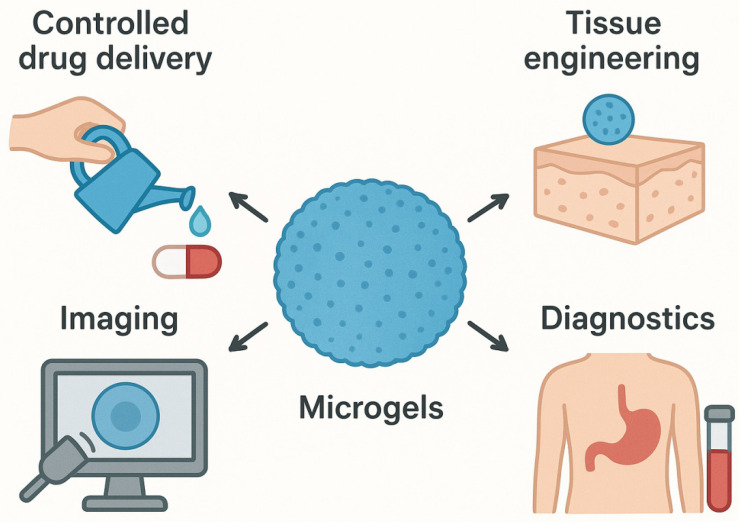
Responsive microgels as versatile platforms for drug delivery, regenerative medicine, imaging, and diagnostics.

Though remarkable progress has been made, significant challenges remain on the path toward clinical translation. Predicting in vivo behavior—such as biodistribution, degradation, immune interactions, and long-term biocompatibility—remains a major barrier. Additionally, achieving scalable, reproducible, and Good Manufacturing Practice (GMP)-compliant manufacturing of microgels is required to meet regulatory expectations for medical products. As responsive microgels move closer to real biomedical use, there is a pressing need to integrate materials innovation with translational considerations. This review provides a comprehensive overview of recent advances in biomedical applications of stimuli-responsive microgels. Emphasis is placed on their multifunctional roles in controlled drug delivery, tissue engineering, imaging, and diagnostics, as shown in Figure 1, while also highlighting emerging opportunities and key challenges associated with translating these dynamic materials toward next-generation biomedical technologies.

## 2. Controlled Drug Delivery

As we mention above, stimuli-responsive microgels are crosslinked hydrogel particles with diameters on the micrometer scale [8]. Within the realm of biomedical applications, stimuli-responsive microgels hold a prominent position due to their highly desirable attributes, including their colloidal nature, porous internal network structure, substantial water content, biocompatibility, tunable sizing, and favorable mechanical characteristics. These outstanding qualities have prompted researchers to engineer a diverse range of stimuli-responsive microgels, each imbued with distinct functionalities, which are aimed at advancing various facets of biomedical applications. Notably, they have played a pivotal role in the development of controlled drug delivery systems [8,9,10,11]. Researchers have explored an array of physical and chemical stimuli, including factors such as heat, light, ultrasound, magnetic fields, pH variations, redox reactions, and enzymatic activity. In response, they have devised a spectrum of responsive microgels meticulously tailored to enable stimuli-triggered or controlled drug release mechanisms [10,12,13]. This section will provide an overview of select examples featuring stimuli-responsive microgels, with a particular focus on those capable of responding to the aforementioned stimuli, illustrating their significance in controlled drug delivery applications.

### 2.1. Temperature-Responsive Microgels

**Figure 2:**
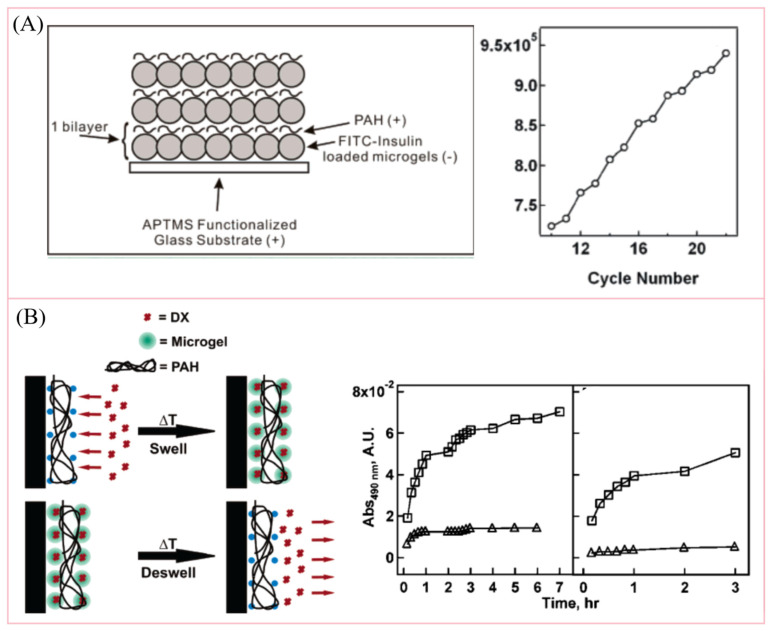
(**A**) Schematic representation of the structure of a three–layer film and cumulative thermally induced release profiles for FITC insulin–loaded microgel thin films over a period of 1 month. Expanded views of cumulative release profiles of cycles 10–22 for the nine–layer films. Reprinted with permission from Ref. [21]. (**B**) Cumulative release profiles for 20–layer films loaded with DX by thermal cycling in an aqueous DX solution (**left**) and cumulative release profiles for 10–layer films temperature cycled between 50 and 20 °C for 1 h at each temperature (squares) and held at 20 °C (triangles) to facilitate release (**right**). Reprinted with permission from Ref. [17].

Among the various microgel types, poly(N-isopropylacrylamide) (pNIPAm)-based microgels have garnered the most extensive research attention to date. These microgels exhibit remarkable thermally reversible behaviors, readily undergoing either swelling or shrinkage as the temperature crosses the lower critical solution temperature (LCST) threshold of 32 °C [14,15,16]. The LCST refers to the temperature below which a polymer is soluble in a given solvent and above which phase separation occurs due to decreased polymer–solvent interactions. In essence, pNIPAm microgels undergo swelling in water when the temperature is below 32 °C, maintaining a fully solvated state. Conversely, they transition into a desolvated globular form when the temperature exceeds 32 °C. Importantly, this conformational shift is entirely reversible and can be repeated multiple times. This distinctive temperature-responsive property of pNIPAm-based microgels has attracted the interest of researchers in the development of temperature-responsive drug delivery platforms [17,18,19,20]. For example, Nolan et al. [21] and Serpe et al. [17] fabricated insulin or doxorubicin-loaded microgels into multi-layer thin films, respectively, and they showcased the ability to precisely control and cyclically modulate payload release by leveraging temperature variations, as shown in Figure 2. Above the LCST, the microgel thin films deswelled, actively releasing drug molecules into the surrounding environment. In contrast, when the temperature fell below the LCST, the films swelled, restricting drug release. Moreover, since the LCST of pNIPAm microgels is close to physiological temperature, pNIPAm-based microgels have also been investigated for in vivo controlled drug delivery applications, as illustrated by Hoare et al. [22].

### 2.2. Light-Responsive Microgels

**Figure 3:**
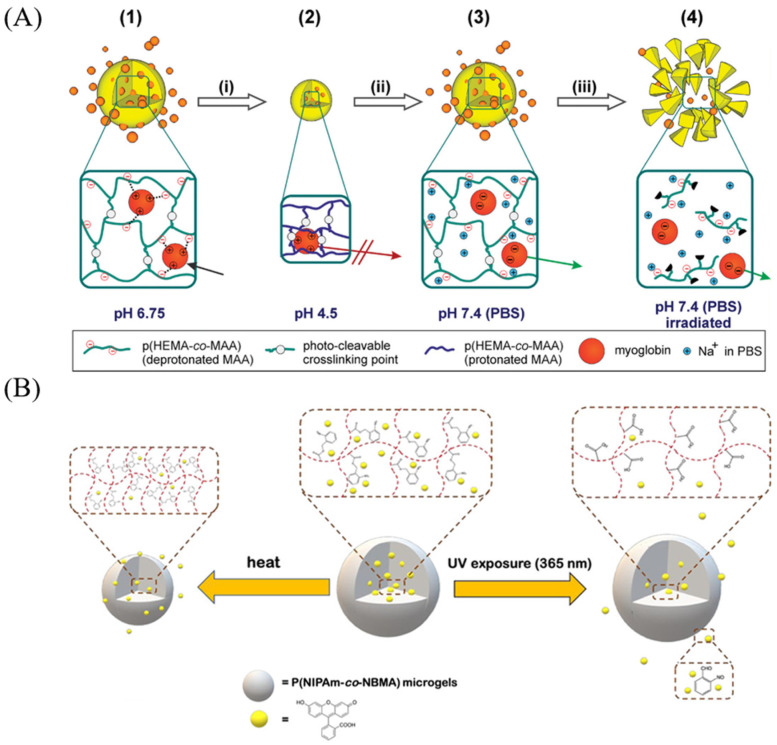
(**A**) Schematic illustration of the loading and release strategy for p(HEMA–*co*–MAA) microgels. (1–2) Loading of large cationic functional compounds into the anionic gel: (i) entrapment by pH–induced deswelling. (3) Diffusion–controlled release: (ii) reswelling in PBS. (4) Degradation-controlled release: (iii) irradiation in PBS. Reprinted with permission from Ref. [25]. (**B**) Schematic of the dual–stimuli–triggered release from fluorescein (“drug”)–loaded pNIPAm–*co*–NBMA microgels in response to temperature and UV light. Reprinted with permission from Ref. [26].

Light stands out as a particularly intriguing stimulus for triggering and controlling drug release from microgels. Its unique physical properties enable remote application with precise control over positioning, intensity, and exposure duration [23]. This capability makes light an ideal choice for initiating drug release from therapeutic delivery vehicles. In most cases, light-responsive microgels were fabricated by incorporating either a light-reversible group (such as azobenzene derivatives) [24] or a light-degradable group (such as O-nitrobenzyl ester derivatives) to induce physical, chemical, and morphological changes to microgels. For example, Klinger and Landfester developed pH- and light- dual-responsive poly(2-hydroxyethyl methacrylate-co-methacrylic acid) p(HEMA-*co*-MAA) microgels for controlled protein release [25]. In their study (Figure 3A), the microgels were formulated with a light-sensitive crosslinker 4-(4-(1-hydroxyethyl)-2-methoxy-5-nitrophenoxy)butan-1-ol (HEMNPB) or 4-(4-(1-hydroxyethyl)-2-methoxy-5-nitrophenoxy)butanoic acid (HEMNPBA), while myoglobin served as the model protein, loaded into the microgels under pH-controlled conditions. Upon exposure to ultraviolet (UV) irradiation at 365 nm, the light-sensitive crosslinker within the microgels underwent a photolysis reaction. This led to the degradation of the crosslinker and, subsequently, the microgels themselves, facilitating the release of the encapsulated protein. In another example, serpe group incorporated O-nitrobenzyl ester-based monomer, O-nitrobenzyl methacrylate (*O*-NBMA) into pNIPAm-based microgels [20,26]. The synthesized p(NIPAm-*co*-NBMA) microgels displayed the ability to encapsulate hydrophobic drugs, such as fluorescein and dexamethasone, primarily through hydrophobic interactions, as shown in Figure 3B. Furthermore, the release of fluorescein could be meticulously regulated by subjecting the p(NIPAm-*co*-NBMA) microgels to external UV exposure at 365 nm. Notably, in the case of dexamethasone release, the liberated drug exhibited the potential to stimulate osteogenic differentiation in human mesenchymal stem cells. These illustrative examples underscore the suitability of light-responsive microgels for precision-controlled drug delivery in biomedical applications.

### 2.3. pH-Responsive Microgels

**Figure 4:**
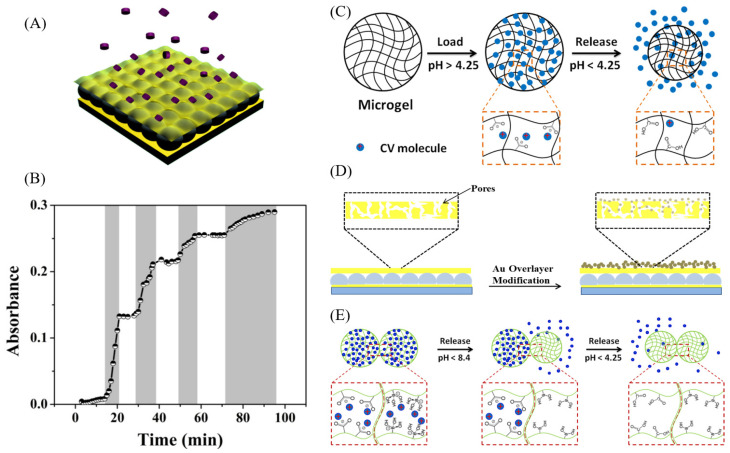
(**A**) Microgel-based reservoir devices were fabricated by sandwiching a monolayer of microgels between two thin gold layers on a glass substrate. (**B**) pH-triggered release profile from a microgel-based reservoir device. (**C**) Loading and release mechanism of CV molecules into and out of pNIPAm-*co*-AAc microgels. Reprinted with permission from Ref. [19]. (**D**) Schematic illustration of pore sizes controlled by tuning surface mophorlogies. Reprinted with permission from Ref. [29]. (**E**) Dual drug release mechanism using different pH-responsive microgels. Reprinted with permission from Ref. [31].

Beyond temperature-responsive microgels, it is possible to engineer microgels with pH-responsive properties. This can be accomplished by introducing specific pH-responsive moieties, such as monomers with pH-dependent protonation states like carboxylic acids and primary amines [27]. Due to the extensive variety of accessible pH-responsive monomers, pH-sensitive microgels with diverse properties have been created for the purpose of controlled drug delivery applications [28,29,30]. Serpe group has been focusing on developing microgel-based reservoir devices as drug delivery platforms for a long time. As demonstrated by Gao et al., the microgel-based reservoir devices were fabricated by sandwiching a monolayer of microgels between two thin gold layers on a glass substrate, as shown in Figure 4 [19,29,31]. In their work, the microgels used were pH-responsive due to the incorporation of acrylic acid (AAc) groups which had a pKa around 4.25. When the pH of the surrounding solution was above its pKa, AAc groups within p(NIPAm-*co*-AAc) microgels were negatively charged due to deprotonation, and in this case, a cationic model drug such as crystal violet could be efficiently loaded into microgels via electrostatic interactions. When the pH of the solution dropped below the pKa of AAc, for example, at pH = 3, the AAc groups and therefore microgels were protonated and the electrostatic interactions between crystal violet molecules and microgels were broken, promoting the release of the model drug. Similarly to the temperature-responsive reversibility, the pH-dependent protonation–deprotonation of microgels was also reversible and could be cycled many times, resulting in pH-dependent “on–off” controlled release of drug molecules.

### 2.4. Other Physical and Chemical Stimuli

In addition to the previously mentioned microgel-based controlled drug delivery systems, there is a significant focus on developing microgels that respond to various other physical and chemical stimuli for drug delivery applications. For instance, the application of a magnetic field as a physical stimulus has garnered substantial attention from researchers, owing to its benefits in terms of controllability, efficiency, and biocompatibility [32,33]. To prepare magnetic field-responsive microgels, magneto-thermoresponsive Fe_3_O_4_ nanoparticles were first synthesized and then incorporated into microgels [34]. Upon the application of an external magnetic field, the magneto-thermo heating transduction effect by Fe_3_O_4_ nanoparticles will generate heat, which results in the heat-induced shrinkage of temperature-responsive microgels, thus triggering drug release within such systems.

In recent years, there has been a growing interest in using glucose levels as a chemical stimulus, primarily due to its crucial role in monitoring the diabetes status of patients and guiding diabetic treatments [35]. Thus, insulin-encapsulated, glucose-responsive microgels were designed and studied for self-regulated insulin release [36]. Typically, these systems involve the incorporation of glucose-sensitive monomers, such as 3-(acrylamido)phenylboronic acid (APBA), into microgels. When exposed to glucose, these microgels undergo conformational changes as a result of the interaction between glucose and APBA moieties, leading to the release of insulin.

Multi-responsive microgels are a cutting-edge solution in the field of controlled drug delivery [37,38]. These versatile microscopic particles are designed to respond to various external stimuli, such as changes in temperature, pH, light, or the presence of specific ions, allowing for highly precise and customized drug release profiles. By intelligently adapting to their surroundings, multi-responsive microgels offer several advantages, including targeted drug delivery, reduced toxicity, improved patient compliance, and the potential for personalized medicine. Their ability to integrate diagnostic and therapeutic functions makes them a promising tool for theranostic applications, advancing the development of more effective and patient-friendly drug delivery systems.

## 3. Tissue Engineering

Cellular behavior and function are strongly influenced by the surrounding microenvironment. In tissue engineering, microgels have attracted considerable interest owing to their excellent biocompatibility and tunable responsiveness to external stimuli. Moreover, there are several advantages to using stimuli-responsive microgels in tissue engineering: (i) Enhanced mechanical properties: Microgels can achieve enhanced mechanical properties by modifying the chemical structure within and between microgels. (ii) Tunable pore size and increased surface area: Microgels offer the ability to tune their pore size and provide a higher internal surface area. This feature provides more adhesion sites for cells and allows for the loading of a greater quantity of drug molecules. (iii) Coupling cell growth and drug delivery: Microgels have the potential to facilitate the coupling of cell growth and infiltration with a drug delivery system. These attributes make stimuli-responsive microgels highly promising for tissue engineering applications [39,40]. Because of these advantages, microgels have been studied in bone tissue [41], cartilage tissue [42], vascular tissue [43], cardiac tissue [44], and neuronal tissue [45].

**Figure 5:**
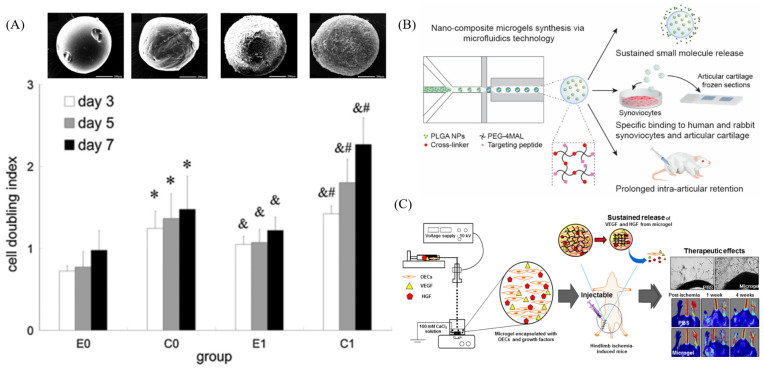
(**A**) SEM micrographs of chitosan microspheres. Emulsion-crosslinked microsphere (E0); coacervate-precipitated microsphere (C0); emulsion-crosslinked microsphere with apatite coating (E1); coacervate-precipitated microsphere with apatite coating (C1). Time-dependent proliferation of MC3T3-E1 cells on different microspheres. * *p* > 0.05, compared to E0. # *p* > 0.05, compared to E1. & *p* > 0.05, compared to the corresponding uncoated microspheres. Reprinted with permission from Ref. [41]. (**B**) Schematic of microgel fabrication. PEG-4MAL was functionalized with targeting peptides and mixed with PLGA NPs prior to microgels’ synthesis via microfluidics technology. Tethering of tissue-targeting peptides to PEG-4MAL microgels provided specific binding to articular cartilage and synoviocytes in vitro. Synoviocyte-targeting PEG-4MAL microgels localized to the synovial membrane in the joint and improved the intra-articular retention time of a model small molecule. Reprinted with permission from Ref. [42]. (**C**) Electrosprayed microgels encapsulating outgrowth endothelial cells (OECs) and growth factors (VEGF and HGF) promoted time-dependent cell proliferation and enhanced viability. Size-controlled microgels enabled sustained growth factor release, supporting angiogenesis in vitro, as shown by tube formation and rat aorta sprouting, and in vivo, with increased vessel formation observed in mice treated with RGD microgels containing OECs and growth factors. Reprinted with permission from Ref. [43].

Bone tissue engineering aims to repair damaged or injured bone tissue structures. In 2013, Shen and co-workers designed a structure using chitosan-based microspheres as an injectable scaffold for bone tissue engineering, which is shown in Figure 5A [41]. They identified chitosan as a suitable material due to its natural origin and favorable properties for promoting osteogenesis. The introduction of an apatite coating on the surface of chitosan microspheres had a positive impact. It improved the attachment, proliferation, and differentiation of osteoblast-like MC3T3-E1 cells by expanding the surface area available for hydroxyapatite interaction, consequently promoting cellular proliferation.

Cartilage tissue is a unique and challenging tissue to regenerate and reconstruct due to its avascular nature and high cell density, which adds complexity to the regenerative process. Castro and co-workers designed a poly (ethylene glycol) nanocomposite microgels for intra-articular injection, which can be used in osteoarthritis treatment [42]. They designed nanocomposite 4-arm-poly (ethylene glycol)-maleimide (PEG-4MAL) with specific peptide and poly(lactic-*co*-glycolic) acid nanoparticles (PLGA NPs), as shown in Figure 5B. The peptides incorporated into the microgel are specifically engineered to bind to cartilage or synoviocytes, allowing the microgel to be recognized and captured by the target tissue. To enhance drug delivery, poly(lactic-*co*-glycolic) acid nanoparticles are employed within the microgel as a small-molecule drug delivery system. This synoviocyte-targeting microgel demonstrated successful localization to the synovial membrane within the joint, thereby improving the intra-articular retention time of the drug.

Endothelial and vascular tissues play a crucial role in facilitating healthy blood flow and the exchange of nutrients and waste gases. The process of angiogenesis is essential for guiding the growth of these tissues. In a study conducted by Kim et al., the authors devised an injectable multifunctional microgel that serves the dual purpose of inhibiting the excessive proliferation of endothelial cells while also promoting neovascularization [43]. The authors used arginine–glycine–aspartic acid (RGD)-conjugated alginate microgels for delivery both cells and growth factors, as shown in Figure 5C. They employed the electrospraying technique to encapsulate outgrowth endothelial cells and growth factors within the microgel. The cells sealed within the microgel exhibited time-dependent proliferation. As the growth factors were gradually released due to their size, the process of angiogenesis was notably enhanced, yielding significant improvements in the desired outcome.

**Figure 6:**
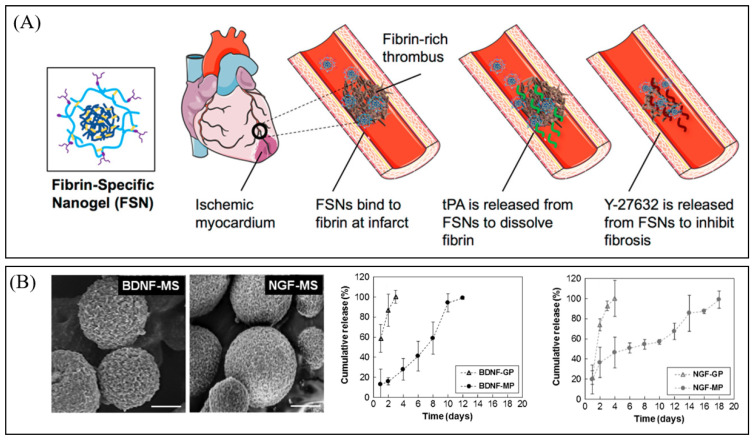
(**A**) Fibrin-specific nanogel design that, when drug-loaded, combats MI, which occurs due to a fibrin-rich thrombus blocking blood flow and creating ischemic myocardium, and subsequent cardiac fibrosis upon reperfusion. Drug-loaded FSNs will bind to fibrin at the infarct site, release a fibrinolytic drug, and release a small-molecule cell contractility inhibitor to mitigate cardiac fibrosis due to reperfusion injury. Reprinted with permission from Ref. [44]. (**B**) SEM images of BDNF-loaded (BDNF-MS) and NGF-loaded (NGF-MS) alginate microspheres and in vitro release of NGF and BDNF. The release profiles of the growth factors from the thermogel (BDNF-GP and NGF-GP) and the microsphere-loaded thermogel (BDNF-MP and NFG-MP) were compared. Reprinted with permission from Ref. [45].

Re-establishing blood flow within the heart poses a significant challenge in regenerating cardiac tissue. Mihalko et al. have created a dual-delivery microgel therapy to address ischemic and fibrotic complications arising from myocardial infarction [44]. They designed pNIPAM nanogels with a fibrin-specific targeting mechanism, forming a dual-delivery system aimed at restoring blood flow and inhibiting cardiac fibrosis post-ischemia–reperfusion injury. These nanogels, with a core of NIPAM and a shell of NIPAM-*co*-AAc, were employed for the targeted delivery of tissue plasminogen activator (tPA) and a cell contractility inhibitor (Y-27632) to the myocardium region. The loaded microgels quickly degraded fibrin and reduced the formation of stress fibers in cardiac cells, as well as the expression of connective tissue growth factor, both of which are elevated in cardiac fibrosis, as illustrated in Figure 6A.

Neuronal tissue engineering is considerably more complex, as it relies on a multitude of factors, encompassing cell types, electrical conduction, growth factor cues, and even cellular orientation. Patel et al. [45] developed a poly (ethylene glycol)-poly (L-alanine) microsphere in polypeptide with brain-derived neurotrophic factor, nerve growth factor and tonsil-derived mesenchymal stem cells, which is shown in Figure 6B. This material exhibits a modulus of 800 Pa at 37 °C, which closely resembles brain tissue. Microgels enable the controlled release of essential growth factors within specific targeted areas.

## 4. Imaging

Stimuli-responsive microgels have garnered growing attention in the field of bioimaging applications, primarily due to their appealing properties. Firstly, their soft and diminutive nature enables them to traverse bodily barriers and reach targeted regions [46]. Secondly, a multitude of water-soluble polymers, such as polyethylene glycol, are non-toxic and biocompatible, which facilitates their utilization in in vivo imaging [47,48,49]. Thirdly, the microgel network serves as a protective barrier, preventing the bioimaging agent from being excreted by cells [46]. Most notably, stimuli-responsive microgels can react to a variety of stimuli, including changes in pH, temperature, and light, leading to enhancements in imaging quality [48]. Thanks to these benefits, microgels find extensive applications in magnetic resonance imaging (MRI) and fluorescence imaging.

**Figure 7:**
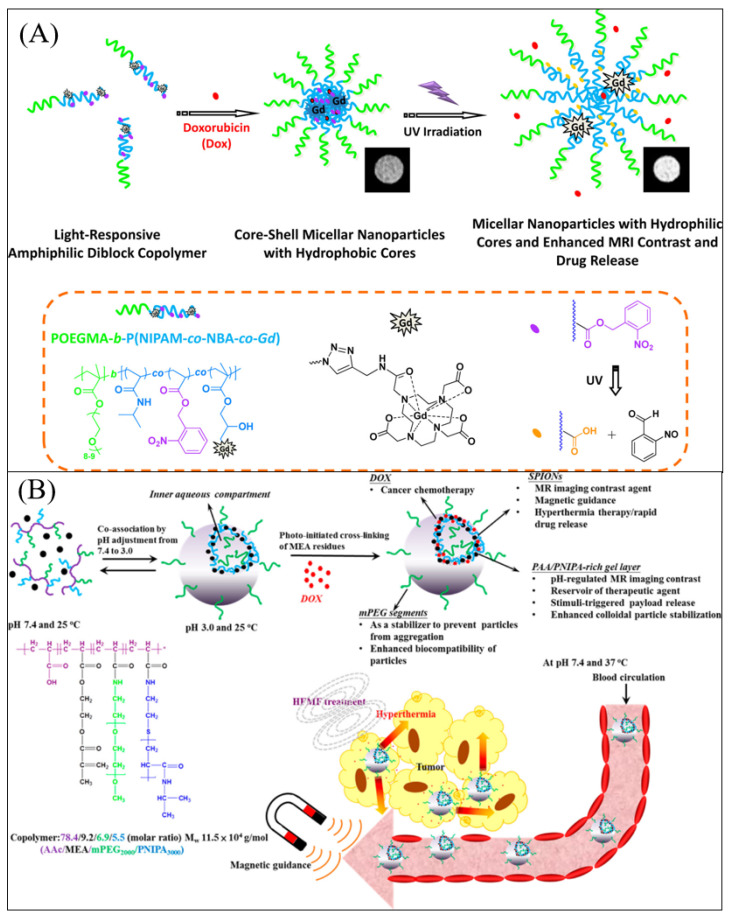
(**A**) Schematic illustration of the fabrication of light-responsive polymeric micelles based on POEGMA-*b*-P(NIPAM-*co*-NBA-*co*-Gd) amphiphilic diblock copolymers, showing light-induced hydrophobic–hydrophilic transition within the micellar core, accompanied by enhanced magnetic resonance (MR) imaging contrast and accelerated release of physically encapsulated hydrophobic drugs (DOX). Reprinted with permission from Ref. [50]. (**B**) Design and construction of DOX-loaded hollow hybrid nanogels as a multifunctional theranostic platform for combined anticancer drug delivery and imaging. Reprinted with permission from Ref. [51].

Magnetic resonance imaging (MRI) is a noninvasive imaging technique used for diagnosing abnormal tissues and organs in clinical applications. Commonly used contrast agents in MRI include Gd^3+^ complexes and superparamagnetic iron oxide nanoparticles (SPIONs), which play a significant role in enhancing MRI performance [46]. A light-triggered polymeric micelle with Gd^3+^ complex was reported by Li et al. [50]. POEGMA-*b*-P(NIPAM-*co*-NBA-*co*-Gd) amphiphilic diblock copolymer was synthesized and self-assembled into micelle particles with Gd^3+^ complex located in the hydrophobic core. Under UV irradiation, the hydrophobic NBA molecules undergo cleavage, leaving behind hydrophilic carboxylic groups, as illustrated in Figure 7A. This transition enables the Gd^3+^ complex to achieve better exchange with water molecules, ultimately enhancing the performance of MRI. In another instance, hollow hybrid nanogels were created by co-assembling superparamagnetic iron oxide nanoparticles (SPIONs) and a graft copolymer. This copolymer consisted of acrylic acid and 2-methacryloylethyl acrylate units forming the backbone, while polyethylene glycol (PEG) and pNIPAm were grafted onto it. Subsequently, these nanogels were crosslinked through photoinitiated reactions involving the 2-methacryloylethyl acrylate residues, as depicted in Figure 7B [51]. The pH-responsive hybrid nanogels exhibited a remarkable increase in transverse relaxivity (r2) value, rising from 138.5 to 265.5 (mM^−1^ s^−1^) as the pH shifted from 4.0 to 7.4. This enhancement was attributed to the deprotonation of acrylic acid, causing the nanogels to swell and facilitating stronger interactions between water molecules and superparamagnetic iron oxide nanoparticles (SPIONs). Furthermore, the gel structure impeded the mobility of water molecules near the SPION surface, extending their interaction time and further improving the MRI performance.

**Figure 8:**
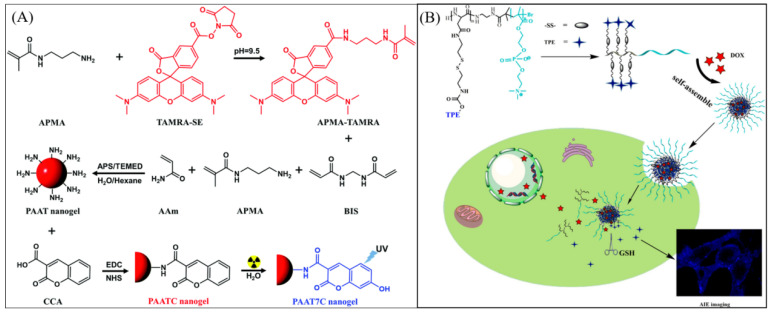
(**A**) Schematic representation of the synthesis and radiation-sensing mechanism of PAATC nanogels for ionizing radiation dose measurement. Reprinted with permission from Ref. [52]. (**B**) Illustration of DOX-loaded TPE-SS-PLAsp-*b*-PMPC micelles, highlighting glutathione (GSH)-responsive drug release and aggregation-induced emission (AIE)-based cellular imaging. Reprinted with permission from Ref. [53].

Fluorescence imaging stands as a widely employed technique for real-time, in vivo bioimaging due to its sensitivity, selectivity, high contrast, cost-effectiveness, and user-friendly operation [46,47,48,49]. Stimuli-responsive microgels, when coupled with fluorescent molecules, are commonly employed in fluorescence bioimaging. For instance, Li et al. developed a micro/nanogel based on polyacrylamide-*co*-*N*-(3-aminopropyl)methacrylamide (PAAm-*co*-APMA) for colorimetric fluorescence assessment of ionizing radiation doses [52]. Two fluorophores, coumarin-3-carboxylic acid (CCA) and 5(6)-carboxytetramethylrhodamine (TAMRA), were covalently linked to the nanogel (PAATC) via an EDC/NHS coupling reaction, as shown in Figure 8A. The fluorescence intensity of the CCA moiety responded to changes in ionizing irradiation doses, while the TAMRA moiety served as a reference fluorophore with its fluorescence intensity remaining unaffected by ionizing irradiation doses. By introducing the nanogel into living human cells (A549), it became possible to measure intracellular ionizing irradiation doses, thus enabling the monitoring of such doses during radiotherapy. In addition, Hu et al. reported a redox-responsive polymeric micelle utilizing aggregation-induced emission (AIE) phenomenon for cell imaging, as illustrated in Figure 8B [53]. The amphiphilic copolymer poly(aspartic acid)-block-poly(2-methacryloyloxyethyl phosphorylcholine) (TPE-SS-PLAsp-*b*-PMPC) incorporated with tetraphenylethene (TPE) could self-assemble into micelles. These micelles possessed a hydrophobic core that constrained the TPE molecules in an aggregated state, leading to the emission of intense blue light. This unique property made them highly suitable for bioimaging applications.

As their advantageous properties have been exploited, stimuli-responsive microgels have emerged as promising candidates for a wide range of bioimaging applications. These microgels have demonstrated exceptional capabilities in various imaging modalities, showcasing their potential to revolutionize the field of medical imaging.

## 5. Diagnostic

The integration of biomolecular sensing with advanced medical and cellular imaging technologies enables real-time detection of specific analytes and efficient signal transduction into interpretable outputs, offering a robust strategy for early disease diagnosis and prompt clinical intervention [54,55,56].

**Figure 9:**
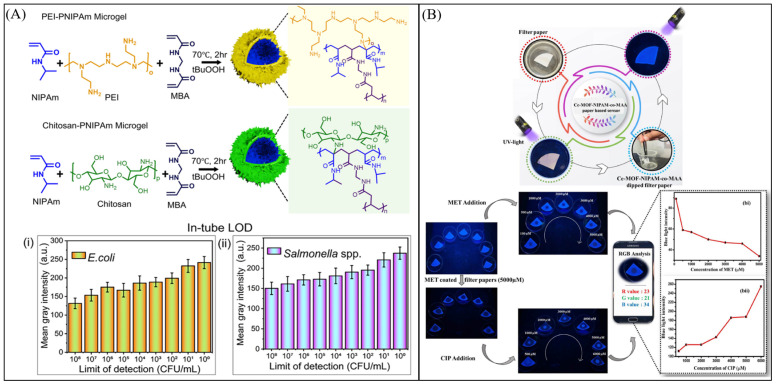
(**A**) Schematic representation of the synthesis of cationic core–shell microgels and the mean gray intensities for the detection range of 10^8^–10^0^ CFU/mL for *E. coli* and *Salmonella* spp. Reprinted with permission from Ref. [68]. (**B**) Schematic representation showing the formation of a paper-based sensor using Ce-MOF-NIPAM-*co*-MAA hybrid microgel solution and representation showing the use of RGB application to determine R, G and B values of the sample along with (**i**,**ii**) showing variation in blue light intensity with concentration of MET and CIP, respectively. Reprinted with permission from Ref. [69].

By integrating stimuli-responsive functionalities—such as sensitivity to pH, temperature, enzymes, redox conditions, light, or specific biomolecules—microgels can effectively convert biological or chemical cues into measurable optical, electrical, or magnetic signals. Owing to their tunable chemistry and soft, porous architectures, functional microgels have been widely explored as sensing elements for the detection of ions, metabolites, proteins, nucleic acids, and disease-associated biomarkers, offering high sensitivity and selectivity. Their deformable networks enable rapid analyte diffusion and signal amplification, while versatile surface modification strategies allow conjugation with recognition ligands, fluorophores, or imaging contrast agents. Moreover, microgels can be engineered for multiplexed detection, real-time monitoring, and seamless integration with imaging modalities or point-of-care diagnostic platforms. Collectively, these features position responsive microgels as promising materials for next-generation diagnostic systems, ranging from biosensors and imaging probes to smart platforms for disease detection and monitoring [48,57,58,59]. Changes in the physical properties of microgels—such as particle size, swelling behavior, fluorescence intensity, or magnetic relaxivity—can be readily detected and quantitatively correlated with the presence or concentration of target analytes, enabling real-time diagnostic readouts [15,60,61,62]. For example, pH- or enzyme-responsive fluorescent microgels have been developed to selectively light up in tumor microenvironments, while redox-responsive microgels can report intracellular glutathione levels through triggered signal changes. Owing to their rapid response, high sensitivity, and facile signal transduction, stimuli-responsive microgels are particularly well suited for integration into point-of-care diagnostic devices, offering portable, fast, and minimally invasive platforms for disease detection and monitoring [63,64,65,66,67]. Jena et al. [68] presented a novel, cost-effective, and field-deployable nucleic-acid detection platform that integrates cationic core–shell microgel systems into a paper-based origami microdevice. The platform enabled rapid and sensitive detection of E. coli and Salmonella spp., achieving an LOD as low as 10 CFU/mL for E. coli and 10^2^ CFU/mL for Salmonella spp. using an MB-based colorimetric readout and validated by RT-PCR analysis, where lower Ct values in microgel systems further confirmed their superior efficiency over polymeric forms, as shown in Figure 9A. Significantly, the device supports multiplex analysis, allowing simultaneous detection of multiple pathogens from a single sample inlet on spatially defined zones, without cross-reactivity or signal interference. Garg et al. reported a novel dual-mode sensor for the sensitive detection of bactericidal agents in real water samples [69]. By integrating porous cerium metal–organic frameworks (Ce-MOF) with PNIPAM-*co*-MAA microgels, the sensor exhibits reversible “turn-off” fluorescence in the presence of MET and “turn-on” fluorescence upon CIP addition. This dynamic, switchable behavior allows ultra-sensitive detection of MET (LOD: 0.16 µM) and CIP (LOD: 0.23 µM), highlighting the sensor’s potential as a reliable tool for environmental antibiotic monitoring, as shown in Figure 9B. For the first time, a microgel-stabilized MOF-based probe has been employed for dual-mode detection via both fluorescence quenching and enhancement. These adaptive hybrid materials exhibited excellent biocompatibility and performed effectively in water analysis, achieving recovery rates between 72% and 145%. To facilitate on-site, instrument-free detection, the hybrid material was incorporated into a low-cost, portable paper-chip sensor, complemented by a smartphone-based color-scanning app and a custom Python algorithm program.

Stimuli-responsive microgels have emerged as versatile diagnostic materials due to their ability to convert biochemical cues into detectable signals. Their tunable physicochemical properties, high surface area, and porous networks enable sensitive recognition of disease-associated analytes, while their compatibility with optical, electrical, and magnetic transduction mechanisms supports real-time and noninvasive diagnostics.

## 6. Conclusions and Perspective

Stimuli-responsive microgels offer tremendous potential across a wide range of biomedical applications due to their unique ability to respond to external stimuli, enabling controlled and targeted delivery of therapeutic agents, biomolecules, and other functional payloads. These versatile materials have demonstrated significant promise not only in drug delivery, tissue engineering, diagnostics, and imaging, but also in gene therapy, wound healing, and medical device coatings.

Looking forward, several key perspectives emerge. Microgels can be precisely tailored to respond to specific physiological conditions, paving the way for personalized treatment strategies and more patient-centric therapies. The development of multi-responsive microgels offers enhanced control over drug release and therapeutic interventions. Additionally, microgels can be combined with diverse therapeutic agents, such as drugs, genes, or proteins, to facilitate combination therapies that target multiple disease mechanisms simultaneously. Integration with nanotechnology, such as nanoparticles or nanocarriers, further expands their capabilities, enabling more efficient, precise, and versatile drug delivery systems.

Despite their promising properties, several limitations of microgels have yet to be addressed for biomedical applications. In particular, their in vivo fate following payload delivery, including degradation, clearance pathways, and potential long-term accumulation, is not yet fully understood. Translating stimuli-responsive microgels from research to clinical practice will require addressing regulatory requirements, ensuring safety, and developing scalable, cost-effective production methods. Collaborative efforts among researchers, clinicians, and regulatory agencies will be essential to advance these materials toward clinical application and broader patient access.

With continued interdisciplinary research and innovation, stimuli-responsive microgels have the potential to revolutionize healthcare by providing more precise, effective, and personalized treatments for a wide range of diseases. Addressing challenges related to safety, regulatory compliance, and large-scale manufacturing will be crucial for their successful integration into clinical practice.

## Data Availability

No new data were created or analyzed in this study.
